# Schizophrenia and pregnancy: a national register-based follow-up study among Finnish women born between 1965 and 1980

**DOI:** 10.1007/s00737-019-0948-0

**Published:** 2019-02-14

**Authors:** Laura Simoila, Erkki Isometsä, Mika Gissler, Jaana Suvisaari, Erja Halmesmäki, Nina Lindberg

**Affiliations:** 1 Psychiatry, Helsinki University and Helsinki University Hospital, Psykiatriakeskus, P.O. Box 590, 00029 HUS, Finland; 2 National Institute for Health and Welfare, Information Services Department, Mannerheimintie 166, 00270 Helsinki, Finland; 3 Research Centre for Child Psychiatry, University of Turku, Lemminkäisenkatu 3, 20520 Turku, Finland; 4 Karolinska Institute, Department of Neurobiology, Care Sciences and Society, Division of Family Medicine, Alfred Nobels allé 23, 14183 Huddinge, Sweden; 5 National Institute for Health and Welfare, Mental Health Unit, P.O.Box 30, 00271 Helsinki, Finland; 6 Femeda-clinic, Kalevankatu 9 A, 00100 Helsinki, Finland; 7 Obstetrics and Gynecology, Helsinki University and Helsinki University Hospital, Naistenklinikka, P.O. Box 140, 00029 HUS, Finland; 8 Forensic Psychiatry, Helsinki University and Helsinki University Hospital, Psykiatriakeskus, P.O. Box 590, 00029 HUS, Finland

**Keywords:** Pregnancy, Conditions related to or aggravated by the pregnancy, Schizoaffective disorder, Schizophrenia, Women

## Abstract

To assess psychosocial and somatic risk factors related to pregnancy, and pregnancy-related complications or disorders in women with schizophrenia compared to population controls. In this register-based cohort study, we identified all Finnish women who were born in 1965–1980 and diagnosed with schizophrenia in psychiatric care before 31 December 2013. For each case, five age- and place-of-birth matched controls were randomly selected. They were followed from the day when the disorder was diagnosed in specialized health care till the end of 2013. The mean follow-up time was 14.0 + 6.91 vs. 14.3 + 6.89 years. Altogether, 1162 singleton pregnancies were found among affected women and 4683 among controls. Affected women were significantly older and more often single; their body mass index before pregnancy was significantly higher, and they smoked significantly more often both in the beginning of pregnancy and after the first trimester than controls. They showed a significantly higher odds for pathologic oral glucose tolerance test (odds ratio (OR) 1.66, 95% confidence interval (95% CI) 1.27–2.17), initiation of insulin treatment (OR 1.84, 95% CI 1.15–2.93), fast fetal growth (OR 1.62, 95% CI 1.03–2.52), premature contractions (OR 2.42, 95% CI 1.31–4.49), hypertension (OR 1.81, 95% CI 1.01–3.27), and pregnancy-related hospitalizations (OR 1.97, 95% CI 1.66–2.33). Suspected damage to the fetus from alcohol/drugs was significantly more common among affected women than controls. Women with schizophrenia have higher prevalence of psychosocial and somatic risk factors related to pregnancy, as well as pregnancy-related complications and disorders than non-affected women.

## Introduction

There has been an increase in pregnancies among women with schizophrenia spectrum disorders (Matevosyan 2011; Solari et al. 2009). For example, in Ontario, Canada, the general fertility rate among women with schizophrenia was 16% higher in 2007–2009 than in 1996–1998 (the incidence rate ratio (IRR) 1.16, 95% confidence interval (CI) 1.04–1.31) (Vigod et al. 2012). However, research focusing on their reproductive health is still scarce and often limited by modest sample sizes. Overall, women with schizophrenia less often have a partner or spouse (Matevosyan 2011) and their pregnancies are more often unplanned than those of healthy women (Matevosyan 2011). Regarding health conditions which increase the risk for problems during pregnancy, women with schizophrenia have a higher risk of obesity and metabolic syndrome (Dipasquale et al. 2013) than the general population. The prevalence of smoking is high in people with schizophrenia (Dalack et al. 1998) and, according to a meta-analysis by Koskinen et al. (2009), approximately, 20% of them have a lifetime diagnosis of alcohol use disorder. Pregnant women with schizophrenia may be more likely to smoke (Bennedsen 1998; Judd et al. 2014) and use alcohol (Bennedsen 1998) and illicit drugs (Judd et al. 2014) than other pregnant women. Pregnancy also appears to worsen the mental health of some women with schizophrenia. As compared to other pregnant women, those with a history of psychosis have reported more anxiety, socio-economical and interpersonal problems, fear about delivery, as well as lack of confidence about their ability to parent (McNeil et al. 1983). Regarding adverse pregnancy outcomes, women with schizophrenia are found to be at higher risk of venous thromboembolism (Ellman et al. 2007), pre-eclampsia/eclampsia (Ellman et al. 2007; Judd et al. 2014; Nguyen et al. 2013; Vigod et al. 2014), and gestational diabetes (Judd et al. 2014; Nguyen et al. 2013) and they have shown a tendency for higher gestational hypertension (Ellman et al. 2007) and increased rates of prenatal hospitalizations (Ellman et al. 2007; Vigod et al. 2014). In contrast, women with schizophrenia have reported receiving less prenatal care than their healthy counterparts (Miller and Finnerty 1996).

Pregnancy and maternity care are influenced by both cultural and socio-economic conditions, as well as the provision and funding of healthcare services. Therefore, research findings may be context-specific and the generalizability of findings between settings, countries, and time periods is uncertain. The purpose of this Finnish register-based national population study was to investigate pregnancy-related health outcomes and complications in women with schizophrenia or schizoaffective disorder. We hypothesized that compared to non-affected women, women with schizophrenia would show higher prevalence of psychosocial and somatic risk factors related to pregnancy, as well as pregnancy-related complications and disorders. Finally, we predicted that, as a consequence of this, women with schizophrenia would have received more intense prenatal care compared to their non-affected counterparts.

## Materials and methods

### Participants

**Fig. 1:**
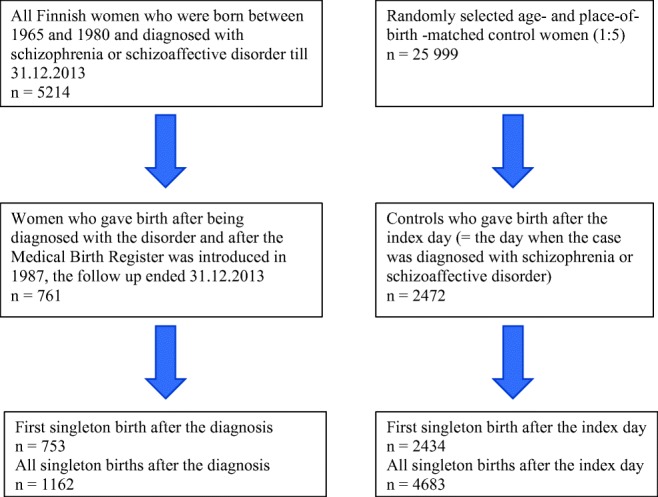
Flow chart of the study project

The sample comprised a Finnish national population of women who were born between Jan. 1, 1965 and Dec. 31, 1980, and diagnosed with schizophrenia or schizoaffective disorder (here, schizophrenia) in specialized health care at some point during the follow-up time ending Dec. 31, 2013 (*N* = 5214). For each woman with schizophrenia, five age- and place-of-birth matched controls were randomly selected from the Finnish Central Population Register (*N* = 25,999). They had not been diagnosed with schizophrenia, schizoaffective disorder or any other psychotic disorder by the end of the follow-up time, but other mental health disorders were allowed. For further information, see Simoila et al. (2018). Flow chart of the sampling of participants is presented in Fig. 1 .

### Diagnoses

We used the Care Register for Health Care of the National Institute of Health and Welfare to obtain the psychiatric diagnoses. In Finland, the International Classification of Diseases—Eighth Revision (ICD-8) (World Health Organization 1965) was used between 1969 and 1986 (schizophrenia 295.0–6, 295.8–9; schizoaffective psychosis 295.7). The third revision of the Diagnostic and Statistical Manual of Mental Disorders (DSM-III-R) (American Psychiatric Association 1987) was used between 1987 and 1995, but the diagnoses were converted to the International Classification of Disease—Ninth Revision (ICD-9) (World Health Organization 1977) codes (schizophrenia: 295.0–6, 295.8–9; schizoaffective psychosis: 295.7). The International Classification of Diseases—Tenth Revision (ICD-10) (World Health organization 1992) has been used since 1996 (schizophrenia, F20; schizoaffective psychosis, F25). The onset of schizophrenia was defined as the day the disorder was diagnosed and coded in specialized health care.

### Follow-up

Women were followed from the onset of the disorder until the individual moved abroad, died, or follow-up ended on Dec. 31, 2013. Death or emigration information was gathered from the Finnish Central Population Register. The mean follow-up time of women with schizophrenia was 14.0 (SD 6.91) years, and, respectively, of controls 14.3 (SD 6.89) years (*p* = 0.001).

### Information on pregnancy

Data were obtained from the Medical Birth Register, maintained by the National Institute of Health and Welfare since 1987. This register covers all delivery hospitals in Finland and includes data on live births and stillbirths of fetuses with a birth weight of at least 500 g or a gestational age of at least 22 weeks, as well as data on the mothers. Healthcare professionals (midwife, nurse, etc.) fill in a data collection form or record the same information electronically. The National Institute of Health and Welfare has the responsibility to check the data quality. It has been reported that most of the register content corresponds well or satisfactorily with medical records. More precisely, in 1991, Gissler et al. (1995) studied the agreement percentage (%) of the Medical Birth Register and medical records and reported the following: mother’s identification number 99.0%, marital status 95.0%, smoking status 93.4%, previous pregnancies 96.3%, previous deliveries 97.4%, gestational age 86.2%, first antenatal visit 94.7%, birth weight 99.0%, 1-min Apgar score 99.5%, and the number of antenatal care visits 62.4%. In this study, all pregnancies leading to singleton births during the follow-up period were included whereas multiple pregnancies were excluded.

We used the following data from the Medical Birth Register: gestational age, parity, maternal age at birth, marital status at the end of the pregnancy, smoking status in the beginning of the pregnancy and after the first trimester (recorded since 1991), weight and height before the pregnancy in order to calculate body mass index, BMI = kg/m^2^ (recorded since 2004), as well as the following pregnancy complications (recorded since 2004): pathological oral glucose tolerance test, initiation of insulin treatment, anemia, and antenatal corticosteroid treatment. The following ICD-10 (World Health organization 1992) diagnoses used for conditions related to or aggravated by the pregnancy (here, pregnancy-related disorders) (recorded since 2004) were collected: hypertension (O13, O16), slow fetal growth (O36.5, P05.0, P05.1, P05.9), fast fetal growth (O36.6), pre-eclampsia (O14), hepatogestosis (O26.6), any vein complication (O22), premature rupture of membrane (O42), oligohydramnios (O41.0), hyperemesis gravidarum (O21.0, O21.1, O21.2, O21.9), urogenital infection (O23), symphyseolysis (O26.7), premature contractions (O47), suspected fetal hypoxia (O36.3), fear of childbirth (O99.80), and suspected fetal injury due to alcohol/drugs (O35.4, O35.5). The data also included variables related to prenatal care: the number of visits to the municipal maternity clinic, the number of outpatient visits to maternity hospital, and hospitalizations related to pregnancy complications or disorders. In Finland, every municipality has its own maternity clinics, which serve mothers-to-be free of charge. Women are advised to contact their local maternity clinic as soon as they find out they are pregnant. The first visit is usually at the gestational age of 8 to 10 weeks. Mothers-to-be with substantial healthcare problems are referred to secondary health care units (outpatient clinics and wards of maternity hospitals).

### Ethics

The Ethics Committee of the Helsinki and Uusimaa Hospital District evaluated the study plan, and the Helsinki University Hospital, and the National Institute of Health and Welfare granted the permission to conduct the study project.

## Data analysis

The analyses were performed in two ways: first, we included each woman’s first singleton pregnancy, which lead to a delivery after she was diagnosed with schizophrenia. Second, we included each woman’s all singleton pregnancies, which led to deliveries after she was diagnosed with schizophrenia. We compared the groups using the chi-square (*χ*^2^) test (cohabiting/married at the end of pregnancy, smoking in the beginning of pregnancy, smoking after the first trimester of pregnancy, pregnancy-related complications and disorders, individuals with one or more hospitalizations), Fisher’s exact test (some pregnancy-related disorders), and independent samples *t* test (age, BMI, parity, number of visits to municipal maternity clinic, number of outpatient visits to maternity hospital). Findings were considered significant when the two-tailed *p* value < 0.05. Coefficient Phi (*x*^2^ test) and Cohen’s *d* were used as measures of effect size. The magnitude of Phi was interpreted as follows: 0.1 as small, 0.3 as moderate, and 0.5 as large effect, and respectively, Cohen’s *d* as follows: 0.2 as small, 0.5 as moderate, and 0.8 as large effect (Cohen 1992).

Next, logistic regression analysis was performed to analyze associations between schizophrenia and pregnancy-related complications and disorders, as well as hospitalizations related to pregnancy during the first pregnancy. With regard to all pregnancies, in order to take into account the clustering of pregnancies within mothers, logistic regression analysis was performed using the generalized estimating equation (GEE) method. Analyses were done unadjusted, but also adjusted models were constructed in order to control for potential confounding factors. Maternal age at birth, marital status (single vs. married/cohabitation), smoking status in the beginning of the pregnancy (yes/no), and parity were chosen to serve as covariates, since all these four variables might independently explain group differences in outcomes, and all had shown good validity in a previous data quality study by Gissler et al. (1995). Variables with less than 10 affected women (in the schizophrenia group, or in the control group, or in both groups) were omitted since such models were considered unstable. Odds ratios (ORs) with 95% CI are reported. Findings were considered significant when the *p* value for the regression model was < 0.05.

Analyses were performed using SPSS 22.0 for Windows and SAS 9.3.

## Results

After the index day, 761 women with schizophrenia and 2472 controls gave birth at least once. Parity measured as the mean number of deliveries was 1.54 (SD 0.92) among women with schizophrenia and 1.92 (SD 0.99) among non-affected women (*t* = − 4.260, *p* < 0.001).

There were altogether 1184 births among women with schizophrenia. We focused on singleton births and identified altogether 1162 (98.1%) singleton births, of which 753 (64.8%) were the first births after the diagnosis. Altogether 205 women had given birth already before they were diagnosed with schizophrenia, but these births (*n* = 359) were not included into the analyses. We restricted the analysis of births in controls to those that occurred after the index day of the case, which led to 4848 births, of which 4683 (96.7%) were singleton births. Of these singleton births, 2434 (52.0%) were the first births after the index day.

### Gestational age of the fetus and woman’s first visit in maternity clinic

Regarding first pregnancies, the mean gestational age of the fetus was 10.4 weeks (SD 4.94) among women with schizophrenia and 9.3 weeks (SD 2.71) among controls (*t* = 5.844, *p* < 0.001). Regarding all pregnancies, the mean gestational age was 10.2 weeks (SD 4.60) among women with schizophrenia and 9.4 (SD 2.73) among controls (*t* = 5.670, *p* < 0.001).

### Delay between the diagnosis and the first visit in maternity clinic

Among women with schizophrenia, the mean delay between the diagnosis and the first visit in maternity clinic was 5.5 (SD 4.20) years when first pregnancies were taken into consideration. Regarding all pregnancies, this delay was 6.8 (SD 4.56) years. The sample included 64 women who were diagnosed with schizophrenia during the pregnancy.

### Psychosocial and somatic risk factors related to pregnancy

Regarding first pregnancies, women with schizophrenia were significantly older during their first pregnancy (Table 1). When all pregnancies were taken into consideration, no statistically significant difference in age was noticed. Regarding both first pregnancies and all pregnancies, women with schizophrenia were more often single at the end of pregnancy compared to their controls. Further, they exhibited significantly higher BMI before pregnancy and smoked significantly more often, both in the beginning of pregnancy and after the first trimester.

**Table 1:** Psychosocial and somatic risk factors related to pregnancy in women with schizophrenia (SZH) and their controls

First pregnancies^*^	SZH (*n* = 753)	Controls (*n* = 2434)	*p* value	ES
Age at birth^a^; mean + SD	30.1 + 5.2	29.2 + 4.3	< 0.001	0.20
Cohabiting/married at the end of pregnancy^a^; *n* (%)	540 (71.7)	2138 (87.8)	< 0.001	0.18^*^
Smoking in the beginning of pregnancy^a^; *n* (%)	274 (36.4)	339 (13.9)	< 0.001	0.24^*^
Smoking after the first trimester of pregnancy^b^; *n* (%)	243 (33.0)	260 (10.9)	< 0.001	0.25^*^
BMI before pregnancy^c^; mean + SD	25.8 + 5.2	24.5 + 4.6	< 0.001	0.27
All pregnancies^*^	SZH (*n* = 1162)	Controls (*n* = 4683)	*p* value	
Age at birth^a^; mean + SD	30.7 + 4.9	30.4 + 4.5	0.104	0.06
Cohabiting/married at the end of pregnancy^a^; *n* (%)	895 (77.0)	4252 (90.8)	< 0.001	0.17^*^
Smoking in the beginning of pregnancy^a^; *n* (%)	408 (35.1)	568 (12.1)	< 0.001	0.25^*^
Smoking after the first trimester of pregnancy^b^; *n* (%)	360 (31.6)	450 (9.8)	< 0.001	0.25^*^
BMI before pregnancy^c^; mean + SD	26.4 + 5.7	24.8 + 4.8	< 0.001	0.30

*SD* standard deviation, *ES* effect size, *BMI* body mass index

*Leading to a delivery after the person was diagnosed with schizophrenia. The chi-square (*χ*^2^) test and independent samples *t* test (age, BMI, parity) were used to compare the groups. Coefficient Phi (*x*^2^ test)^*^ and Cohen’s *d* (independent samples *t* test) were used as effect size measures

^a^Recorded since 1987

^b^Recorded since 1991

^c^Recorded since 2004

### Pregnancy-related complications and disorders

Regarding both first pregnancies and all pregnancies, women with schizophrenia exhibited significantly more often than controls pathologic glucose tolerance test, initiation of insulin treatment and anemia (Table 2). Further, suspected fetal injury due to alcohol/drugs was significantly more prevalent among women with schizophrenia than among controls. Regarding first pregnancies, women with schizophrenia suffered significantly more often than controls from premature contractions and fast fetal growth. However, when all pregnancies were taken into consideration, differences in these variables were not statistically significant. With regard to all pregnancies, exhaustion was significantly more prevalent among women with schizophrenia than among controls. However, when only first pregnancies were taken into consideration, no statistically significant difference in exhaustion was observed.

**Table 2:** Conditions related to or aggravated by the pregnancy^a^ in women with schizophrenia (SZH) and their controls

First pregnancies^*^; *n* (%)	SZH (*n* = 753)	Controls (*n* = 2434)	*p* value	ES
Pathologic oral glucose tolerance test	75 (10.2)	115 (4.8)	< 0.001	0.28
Initiation of insulin treatment	17 (2.3)	27 (1.1)	0.018	0.18
Anemia	12 (1.6)	15 (0.6)	0.001	0.27
Antenatal corticosteroid treatment	8 (1.1)	19 (0.8)	0.461	< 0.10
Premature contractions	17 (2.0)	30 (1.3)	0.041	0.11
Hypertension	15 (2.0)	33 (1.4)	0.210	< 0.10
Suspected fetal injury due to alcohol/drugs	9 (2.4)	1 (0.04)	< 0.001	NA
Fast fetal growth	8 (1.1)	8 (0.3)	0.013	< 0.10
Slow fetal growth	7 (0.9)	12 (0.5)	0.174	< 0.10
Fear of childbirth	6 (0.8)	12 (0.5)	0.331	NA
Pre-eclampsia	6 (0.8)	26 (1.1)	0.514	NA
Premature rupture of membrane	4 (0.5)	21 (0.9)	0.482	NA
Hepatogestosis	3 (0.4)	8 (0.3)	0.728	NA
Oligohydramnios	2 (0.1)	12 (0.5)	0.541	NA
Any vein complication	2 (0.1)	1 (0.04)	0.141	NA
Exhaustion	2 (0.2)	2 (0.1)	0.239	NA
Hyperemesis gravidarum	1 (0.1)	2 (0.1)	0.555	NA
Urogenital infection	1 (0.1)	2 (0.1)	0.555	NA
Symphyseolysis	1 (0.1)	1 (0.04)	0.417	NA
All pregnancies^*^; *n* (%)	SZH (*n* = 1162)	Controls (*n* = 4683)	*p* value	ES
Pathologic oral glucose tolerance test	132 (11.6)	290 (6.3)	< 0.001	0.29
Initiation of insulin treatment	35 (3.1)	69 (1.5)	< 0.001	0.19
Anemia	22 (1.9)	44 (1.0)	0.006	0.12
Antenatal corticosteroid treatment	11 (1.0)	34 (0.7)	0.441	< 0.10
Premature contractions	24 (2.1)	78 (1.7)	0.351	< 0.10
Hypertension	18 (1.6)	57 (1.2)	0.368	< 0.10
Suspected fetal injury due to alcohol/drugs	12 (1.1)	2 (0.04)	< 0.001	NA
Slow fetal growth	11 (1.0)	24 (0.5)	0.086	< 0.10
Fast fetal growth	11 (1.0)	23 (0.5)	0.068	< 0.10
Fear of childbirth	9 (0.8)	34 (0.7)	0.863	< 0.10
Pre-eclampsia	7 (0.6)	47 (1.0)	0.201	< 0.10
Exhaustion	7 (0.6)	7 (0.2)	0.005	0.19
Hepatogestosis	6 (0.5)	23 (0.5)	0.913	NA
Premature rupture of membrane	5 (0.4)	34 (0.7)	0.319	NA
Any vein complication	3 (0.3)	5 (0.1)	0.200	NA
Oligohydramnios	2 (0.2)	16 (0.3)	0.554	NA
Hyperemesis gravidarum	2 (0.2)	2 (0.04)	0.179	NA
Urogenital infection	2 (0.2)	6 (0.1)	0.663	NA
Symphyseolysis	2 (0.2)	3 (0.06)	0.287	NA
Suspected fetal hypoxia	1 (0.1)	0 (0.0)	0.199	NA

*ES* effect size, *NA* not applicable

*Leading to a delivery after the person was diagnosed with schizophrenia. The chi-square (*χ*^2^) test and Fisher’s exact test was used to compare the groups. Coefficient Phi (*x*^2^ test) was used as an effect size measure

^a^Recorded since 2004

### Prenatal care

Regarding both first pregnancies and all pregnancies, the number of municipal maternity clinic visits as well as the number of visits to maternity hospital policlinics was significantly higher among women with schizophrenia than among controls (Table 3). Also, the proportion of women with one or more pregnancy-related hospitalizations was significantly higher among women with schizophrenia than among controls.

**Table 3:** Prenatal care^a^ among women with schizophrenia (SZH) and their controls

First pregnancies*	SZH (*n* = 753)	Controls (*n* = 2434)	*p* value	ES
Number of visits to municipal maternity clinic; mean + SD	17.4 + 6.9	16.8 + 5.5	0.005	0.11
Number of outpatient visits to maternity hospital; mean + SD	4.2 + 3.1	2.9 + 2.8	< 0.001	0.45
Persons with one or more hospitalizations; *n* (%)	235 (31.2)	464 (19.1)	< 0.001	0.13^*^
All pregnancies*	SZH (*n* = 1162)	Controls (*n* = 4683)	*p* value	
Number of visits in municipal maternity clinic; mean + SD	17.3 + 6.8	16.5 + 5.5	< 0.001	0.12
Number of outpatient visits to maternity hospital; mean + SD	4.1 + 3.2	2.9 + 2.8	< 0.001	0.42
Persons with one or more hospitalizations; *n* (%)	332 (28.6)	809 (17.3)	< 0.001	0.11^*^

*SD* standard deviation, *ES* effect size

*Leading to a delivery after the person was diagnosed with schizophrenia. The independent samples *t* test (visits) and chi-square test (*χ*^2^) were used to compare the groups. Coefficient Phi (*x*^2^ test)^*^ and Cohen’s *d* (independent samples *t* test) were used as effect size measures

^a^Recorded since 1987

### Associations between schizophrenia and pregnancy-related complications/disorders and hospitalizations

Regarding first pregnancies, in unadjusted model, women with schizophrenia exhibited a 2-fold increased risk of pathological oral glucose tolerance test, an over 2-fold increased risk of anemia, and an almost 2-fold increased risk of being hospitalized at least once because of pregnancy-related complications or disorders (Table 4). In adjusted model, women with schizophrenia showed an almost 2-fold increased risk of pathological oral glucose tolerance test and hypertension and an over 2-fold increased risk of premature contractions. Their risk of hospitalization was over 2-fold higher. Regarding all pregnancies, in unadjusted model, women with schizophrenia exhibited an almost 2-fold risk of pathologic oral glucose tolerance test and anemia. Their risk of hospitalization was almost 2-fold. In adjusted model, women with schizophrenia showed an almost 2-fold increased risk of pathologic oral glucose tolerance test, initiation of insulin treatment, and fast fetal growth. Their risk of hospitalization was almost 2-fold higher.

**Table 4:** The risk of pregnancy-related complications, disorders, and hospitalizations among women with schizophrenia when controls served as a reference group

First pregnancies*	Unadjusted model, OR (95% CI)	Adjusted model, OR (95% CI)
Pathologic oral glucose tolerance test	2.00 (1.43–2.82)^a^	1.75 (1.24–2.46)^a^
Initiation of insulin treatment	1.09 (0.55–2.12)	1.51 (0.78–2.92)
Anemia	2.23 (1.02–4.86)^a^	2.25 (0.99–5.10)
Premature contractions	1.68 (0.92–3.10)	2.42 (1.31–4.49)^a^
Hypertension	1.15 (0.61–2.15)	1.81 (1.01–3.27)^a^
Individuals with one or more hospitalizations	1.91 (1.58–2.30)^a^	2.12 (1.73–2.58)^a^
All pregnancies*	Unadjusted model, OR (95% CI)	Adjusted model, OR (95% CI)
Pathologic oral glucose tolerance test	1.79 (1.40–2.28)^a^	1.66 (1.27–2.17)^a^
Initiation of insulin treatment	1.32 (0.84–2.08)	1.84 (1.15–2.93)^a^
Anemia during pregnancy	1.82 (1.08–3.09)^a^	1.66 (0.94–2.94)
Antenatal corticosteroid treatment	1.14 (0.57–2.28)	0.95 (0.47–1.90)
Premature contractions	1.10 (0.57–2.28)	1.28 (0.78–2.10)
Hypertension	1.02 (0.59–1.76)	1.53 (0.91–2.56)
Fast fetal growth	1.92 (0.93–3.96)	1.62 (1.03–2.52)^a^
Slow fetal growth	1.84 (0.90–3.78)	1.53 (0.91–2.56)
Individuals with one or more hospitalizations	1.91 (1.65–2.22)^a^	1.97 (1.66–2.33)^a^

*OR* odds ratio, *CI* confidence interval

*Leading to a delivery after the person was diagnosed with schizophrenia. Results of logistic regression and generalized estimating equation (GEE) models are provided. Maternal age at birth, marital status (dichotomous: single vs. married or cohabitation), smoking status in the beginning of the pregnancy (yes vs. no), and parity were used as covariates

^a^Statistically significant finding

## Discussion

This register-based national population study comprised all pregnancies of women born in 1965–1980 who were diagnosed with schizophrenia in Finland. As we hypothesized, women with schizophrenia showed a higher prevalence of pregnancy-related psychosocial and somatic risk factors, as well as pregnancy-related complications and disorders than their non-affected counterparts. They had also received more intense prenatal care than their controls. Overall, findings related to first pregnancies and those related to all pregnancies were much alike. Thus, it seems that risk factors and problems related to pregnancy persist irrespective of parity among women with schizophrenia.

Regarding risk factors, women with schizophrenia showed significantly higher BMI before pregnancy. The finding was expected, since among patients with schizophrenia, obesity is approximately twice as prevalent as in the general population (Allison et al. 2009). This is mostly due to antipsychotic medications, unhealthy diet, and sedentary lifestyle (Manu et al. 2015). Negative symptoms of schizophrenia including apathy, lack of drive and volition, and withdrawal from social interaction can hamper a patient’s ability to take care of herself, as well as to reduce her motivation to participate activities. Lifestyle interventions that target diet, exercise, and behavior are important for prevention and treatment of being overweight or obese (Ward et al. 2015). This is also true for individuals with severe mental illness: a recent meta-analysis of 25 lifestyle interventions showed large effect sizes in weight gain prevention and moderate effect sizes in weight loss studies (Bruins et al. 2014). We found that women with schizophrenia smoked significantly more often in the beginning of pregnancy, as well as after the first trimester than the unexposed women. Poverty and low education level (Tidey and Miller 2015), the self-medication hypothesis (Rüther et al. 2014), and common genetic pathways (Loukola et al. 2014) may explain the strong relationship between nicotine dependency and schizophrenia. However, individuals with schizophrenia are interested in and capable of smoking cessation (Dickerson et al. 2013; Gilbody et al. 2015). In summary, our findings underline the need for targeted health education and lifestyle interventions for women with schizophrenia who plan pregnancy or have become pregnant, and possibly also for those at risk for unplanned pregnancy.

As in recent studies (Judd et al. 2014; Nguyen et al. 2013), affected women with their first pregnancies showed a 1.5-fold increased risk of pathologic oral glucose tolerance test. In all pregnancies, with adjustment for the clustering of pregnancies within mothers, women with schizophrenia showed almost a 2-fold increased risk of both pathological oral glucose tolerance test and initiation of insulin treatment. Furthermore, in accordance with Vigod et al. (2014), their risk of fast fetal growth was approximately 1.6-fold higher. This is probably explained by maternal hyperglycemia, since offsprings of mothers with gestational diabetes have higher weights at birth and later in life (Baptiste-Roberts et al. 2012). A recent meta-analysis on nutrition interventions among individuals with a severe mental disorder demonstrated that significant improvements are achievable in blood glucose levels, especially when the intervention is led by a dietician (Teasdale et al. 2017). Although many antipsychotics are known to increase the risk of gestational diabetes, women with schizophrenia are not recommended to discontinue their medication during pregnancy (McCauley-Elsom et al. 2010) since relapse rates are high (Spielvogel and Lee 2010) and untreated psychosis is a serious risk for the fetus (Einarson 2010; Jablensky et al. 2005). The most up-to-date recommendation is to avoid polypharmacy, use the lowest effective dose, and provide close monitoring (Seeman 2013). If there are risk factors for gestational diabetes, olanzapine should be avoided unless the patient’s history suggests that a switch to another medication significantly enhances her risk of recurrence (Barnes and Schizophrenia Consensus Group of British Association for Psychopharmacology 2011). In the case of clozapine, concerns about the potential for relapse usually outweigh concerns about its dysglycemic effect (Barnes et al. 2011).

In contrast to previous studies (Ellman et al. 2007; Judd et al. 2014; Nguyen et al. 2013; Vigod et al. 2014), we found no group difference in pre-eclampsia/eclampsia. Regarding first pregnancies, however, the risk of gestational hypertension was almost 2-fold higher in women with schizophrenia. Gestational hypertension is related to nulliparity, as well as to overweight status and hyperglycemia (Shen et al. 2017) which probably explains our finding. The risk of premature contractions was more than 2-fold higher in schizophrenic women with their first pregnancies. Psychosocial factors like neuroticism (Handelzalts et al. 2016), low education level, poor relationships with others, as well as impaired coping skills (Facchinetti et al. 2007) have all been associated with premature contractions, which might, at least partly, explain this finding. Regarding all pregnancies, the diagnosis of gestational exhaustion was substantially more frequent among women with schizophrenia than among non-affected women. These findings highlight that psychosocial interventions to increase psychological well-being and environmental mastery should be actively offered to pregnant women with schizophrenia.

Schizophrenia and substance use disorders frequently co-occur (Kessler et al. 2005). In this study, maternal care for (suspected) harm to the fetus from alcohol and/or drugs was fortunately rare, but substantially more common among women with schizophrenia than among non-affected women. Our finding underlines that the monitoring of pregnant women with schizophrenia should include substance use screening. When necessary, treatment of concomitant substance misuse, and schizophrenia should be integrated. The recommended psychosocial treatments include motivational interviewing, psychoeducation, and cognitive behavioral therapy (The Finnish Medical Society Duodecim and the Finnish Psychiatric Association 2015).

In line with earlier findings (Ellman et al. 2007; Vigod et al. 2014), we found that the number of visits to maternity clinics and outpatient clinics of maternity hospitals, as well as the proportion of women with one or more hospitalizations related to pregnancy complications or disorders were significantly higher among women with schizophrenia than among non-affected women. In this perspective, pregnancies of women with schizophrenia could be seen as challenging. These women have both psychosocial and somatic problems and, because of this, intense collaboration between mental health providers, social workers, gynecologists, and obstetrics is needed. Psychiatrists must be prepared to adjust antipsychotic doses frequently, as per clinical status (Seeman 2013). Psychoeducation can reduce the risks of pregnancy complications for women with schizophrenia and short-term focused psychotherapy can be useful for some pregnant women with schizophrenia (Solari et al. 2009). If support networks around a mother-to-be with schizophrenia are lacking, social services can provide supportive interventions. The Finnish child welfare law was renewed in 2010; an anticipatory child welfare notification can be made already during pregnancy if there is a reason to assume that the newborn is going to need actions of child welfare. The pregnancy-related complications and disorders should be screened and treated as well as possible, and it is suggested that mothers-to-be with schizophrenia should be educated regarding signs of labor and familiarized in advance with the setting in which the birth will take place (Seeman 2013). Further research on maternal and fetal outcomes of childbirth is also important.

### Strengths and limitations

The strengths of this study include our ability to investigate the Finnish national population of women with schizophrenia or schizoaffective disorder, the relatively long follow-up time, and the good-quality Finnish health registers (Aro et al. 1990; Gissler et al. 1995). Also, the diagnoses of psychotic disorders have been shown to be reliable (Isohanni et al. 1997; Pihlajamaa et al. 2008). However, some limitations need to be considered: first, we used an age- and place-of-birth matched control group for comparison, but confounding factors such as socioeconomic status were not taken into account. This might have affected outcomes such as nutrition and body weight. In Finland, most patients with schizophrenia are on disability pension (Perälä et al. 2008). However, the municipal healthcare services are funded by tax revenues and are available to all citizens. Second, we were limited to variables that were recorded in the national registers described earlier. Unfortunately, we had no information about women’s non-psychotic psychopathology, medications prescribed to the women, utilization of mental health services, or the nature or amount of their substance use. Women with schizophrenia showed higher prevalence of psychosocial and somatic risk factors related to pregnancy, as well as higher prevalence of pregnancy-related complications and disorders as compared with their non-affected counterparts, but it might also be that they were offered more intense prenatal care because of the psychiatric disorder per se. On the other hand, we can verify that despite their serious mental health disorder, women with schizophrenia are able to use these services. Third, there may also be differences between individual clinicians and local customs within hospitals in the diagnosis and reportage of ICD-10 diagnoses related to childbirth. Fourth, we assumed that the onset of schizophrenia was the day the disorder was diagnosed in specialized health care and we had no information about psychotic symptoms before this date. Neither had we information about women’s psychotic symptoms during pregnancy. Fourth, the Medical Birth Register was introduced in 1987, which means that our data did not comprise pregnancies before this. Fifth, considering the high number of outcomes, some of the observed associations may have occurred by chance. Also, in bivariate analyses, effect sizes showed only weak or moderate associations. Finally, the generalizability of findings to other countries and settings need to be examined. In particular, findings related to service use may vary depending on models of provision and funding of services.

## Conclusions

Women with schizophrenia show higher prevalence of psychosocial and somatic risk factors related to pregnancy, as well as conditions related to and aggravated by the pregnancy than their non-affected counterparts. Targeted health education and lifestyle interventions, as well as close collaboration between mental health professionals, social workers, gynecologists, and obstetricians are needed.

## References

[CR1] Allison DB, Newcomer JW, Dunn AL, Blumenthal JA, Fabricatore AN, Daumit GL, Daumit GL, Cope MB, Riley WT, Vreeland B, Hibbeln JR, Alpert JE (2009). Obesity among those with mental disorders: A National Institute of Mental Health meeting report. Am J Prev Med.

[CR2] American Psychiatric Association (1987). Diagnostic and statistical manual of mental disorders (3^rd^ ed., Revised).

[CR3] Aro S, Koskinen R, Keskimäki I (1990). Reliability of hospital discharge data concerning diagnosis. treatments and accidents Duodecim.

[CR4] Baptiste-Roberts K, Nicholson WK, Wang NY, Brancati FL (2012). Gestational diabetes and subsequent growth patterns of offspring: the National Collaborative Perinatal Project. Matern Child Health J.

[CR5] Barnes TR, Schizophrenia Consensus Group of British Association for Psychopharmacology (2011). Evidence-based guidelines for the pharmacological treatment of schizophrenia: recommendations from the British Association for Psychopharmacology. J Psychopharmacol.

[CR6] Bennedsen BE (1998). Adverse pregnancy outcome in schizophrenic women: occurrence and risk factors. Schizophr Res.

[CR7] Bruins J, Jörg F, Bruggeman R, Slooff C, Corpeleijn E, Pijnenborg M (2014). The effects of lifestyle interventions on (long-term) weight management, cardiometabolic risk and depressive symptoms in people with psychotic disorders: a meta-analysis. PLoS One.

[CR8] Cohen J (1992). A power primer. Psychol Bull.

[CR9] Dalack W, Healy D, Meador-Woodruff JH (1998). Nicotine dependence in schizophrenia: clinical phenomena and laboratory findings. Am J Psychiatry.

[CR10] Dickerson F, Stallings CR, Origoni AE, Vaughan C, Khushalani S, Schroeder J, Yolken RH (2013). Cigarette smoking among persons with schizophrenia or bipolar disorder in routine clinical settings, 1999-2011. Psychiatr Serv.

[CR11] Dipasquale S, Pariante CM, Dazzan P, Aguglia E, McGuire P, Mondelli V (2013). The dietary pattern of patients with schizophrenia: a systematic review. J Psychiatr Res.

[CR12] Einarson A (2010). Antipsychotic medication (safety/risk) during pregnancy and breastfeeding. Curr Womens Health Rev.

[CR13] Ellman LM, Huttunen M, Lönnqvist J, Cannon TD (2007). The effects of genetic liability for schizophrenia and maternal smoking during pregnancy on obstetrical complications. Schizophr Res.

[CR14] Facchinetti F, Ottolini F, Fazzio M, Rigatelli M, Volpe A (2007). Psychosocial factors associated with preterm uterine contractions. Psychother Psychosom.

[CR15] Gilbody S, Peckham E, Man M, Mitchell N, Li J, Becque T (2015). Bespoke smoking cessation for people with severe mental ill health (SCIMITAR): a pilot randomised controlled trial. Lancet Psychiatry.

[CR16] Gissler M, Teperi J, Hemminki E, Merilainen J (1995). Data quality after restructuring a national medical registry. Scand J Soc Med.

[CR17] Handelzalts JE, Krissi H, Levy S, Freund Y, Carmiel N, Ashwal E, Peled Y (2016). Personality, preterm labor contractions, and psychological consequences. Arch Gynecol Obstet.

[CR18] Isohanni M, Mäkikyrö T, Moring J, Räsänen P, Hakko H, Partanen U (1997). A comparison of clinical and research DSMIII-R diagnoses of schizophrenia in a Finnish national birth cohort. Clinical and research diagnoses of schizophrenia. Soc Psychiatry Psychiatr Epidemiol.

[CR19] Jablensky AV, Morgan V, Zubrick SR, Bower C, Yellachich LA (2005). Pregnancy, delivery, and neonatal complications in a population cohort of women with schizophrenia and major affective disorders. Am J Psychiatry.

[CR20] Judd F, Komiti A, Sheehan P, Newman L, Castle D, Everall I (2014). Adverse obstetric and neonatal outcomes in women with severe mental illness: to what extent can they be prevented?. Schizophr Res.

[CR21] Kessler RC, Berglund P, Demler O, Jin R, Merikangas KR, Walters EE (2005). Lifetime prevalence and age-of-onset distributions of DSM-IV disorders in the National Comorbidity Survey Replication. Arch Gen Psychiatry.

[CR22] Koskinen J, Lohonen J, Koponen H, Isohanni M, Miettunen J (2009). Prevalence of alcohol use disorders in schizophrenia-a systematic review and meta-analysis. Acta Psychiatr Scand.

[CR23] Loukola A, Wedenoja J, Keskitalo-Vuokko K, Broms U, Korhonen T, Ripatti S, Sarin AP, Pitkäniemi J, He L, Häppölä A, Heikkilä K, Chou YL, Pergadia ML, Heath AC, Montgomery GW, Martin NG, Madden PAF, Kaprio J (2014). Genome-wide association study on detailed profiles of smoking behavior and nicotine dependence in a twin sample. Mol Psychiatry.

[CR24] Manu P, Dima L, Shulman M, Vancampfort D, De Hert M, Correll CU (2015). Weight gain and obesity in schizophrenia: epidemiology, pathobiology, and management. Acta Psychiatr Scand.

[CR25] Matevosyan NR (2011). Pregnancy and postpartum specifics in women with schizophrenia: a meta-study. Arch Gynecol Obstet.

[CR26] McCauley-Elsom K, Gurvich C, Elsom SJ, Kulkarni J (2010). Antipsychotics in pregnancy. J Psychiatr Ment Health Nurs.

[CR27] McNeil TF, Kaij L, Malmquist-Larsson A (1983). Pregnant women with nonorganic psychosis: life situation and experience of pregnancy. Acta Psychiatr Scand.

[CR28] Miller LJ, Finnerty M (1996). Sexuality, pregnancy and childrearing among women with schizophrenia spectrum disorders. Psychiatr Serv.

[CR29] Nguyen TN, Faulkner D, Frayne JS, Allen S, Hauck YL, Rock D, Rampono J (2013). Obstetric and neonatal outcomes of pregnant women with severe mental illness at a specialist antenatal clinic. Med J Aust.

[CR30] Perälä J, Saarni SI, Ostamo A, Pirkola S, Haukka J, Härkänen T (2008). Geographic variation and sociodemographic characteristics of psychotic disorders in Finland. Schizophr Res.

[CR31] Pihlajamaa J, Suvisaari J, Henriksson M, Heilä H, Karjalainen E, Koskela J, Cannon M, Lönnqvist J (2008). The validity of schizophrenia diagnosis in the Finnish Hospital Discharge Register: findings from a 10-year birth cohort sample. Nord J Psychiatry.

[CR32] Rüther T, Bobes J, De Hert M, Svensson TH, Mann K, Batra A (2014). EPA guidance on tobacco dependence and strategies for smoking cessation in people with mental illness. Eur Psychiatry.

[CR33] Seeman MV (2013). Clinical interventions for women with schizophrenia: pregnancy. Acta Psychiatr Scand.

[CR34] Shen M, Smith GN, Rodger M, White RR, Walker MC, Wen SW (2017). Comparison of risk factors and outcomes of gestational hypertension and pre-eclampsia. PLoS One.

[CR35] Simoila L, Isometsä E, Gissler M, Suvisaari J, Sailas E, Halmesmäki E, Lindberg N (2018). Schizophrenia and induced abortions: a national register-based follow-up study among Finnish women born between 1965-1980 with schizophrenia or schizoaffective disorder. Schizophr Res.

[CR36] Solari H, Dickson KE, Miller L (2009). Understanding and treating women with schizophrenia during pregnancy and postpartum—Motherisk Update 2008. Can J Clin Pharmacol.

[CR37] Spielvogel AM, Lee EK (2010). Indication for psychiatric inpatient hospitalization for pregnant psychotic women. Curr Womens Health Rev.

[CR38] Teasdale SB, Ward PB, Rosenbaum S, Samaras K, Stubbs B (2017). Solving a weighty problem: systematic review and meta-analysis of nutrition interventions in severe mental illness. Br J Psychiatry.

[CR39] The Finnish Medical Society Duodecim and the Finnish Psychiatric Association (2015) Current Care Guidelines for Schizophrenia. Available at: http://www.kaypahoito.fi. Accessed 6 Oct 2017

[CR40] Tidey JW, Miller ME (2015). Smoking cessation and reduction in people with chronic mental illness. BMJ.

[CR41] Vigod SN, Seeman MV, Ray JG, Anderson GM, Dennis CL, Grigoriadis S (2012). Temporal trends in general and age-specific fertility rates among women with schizophrenia (1996-2009): a population-based study in Ontario, Canada. Schizophr Res.

[CR42] Vigod SN, Kurdyak PA, Dennis CL, Gruneir A, Newman A, Seeman MV, Rochon PA, Anderson GM, Grigoriadis S, Ray JG (2014). Maternal and newborn outcomes among women with schizophrenia: a retrospective population-based cohort study. BJOG.

[CR43] Ward M, White D, Druss B (2015). A meta-review of lifestyle interventions for cardiovascular risk factors in the general medical population: lessons for individuals with serious mental illness. J Clin Psychiatry.

[CR44] World Health Organization (1965). Manual of the international statistical classification of diseases, injuries, and causes of death, 8^th^ revision. (ICD-8).

[CR45] World Health Organization (1977). Manual of the international statistical classification of diseases, injuries, and causes of death, 9^th^ revision (ICD-9).

[CR46] World Health Organization (1992). International statistical classification of diseases and health related problems, 10^th^ revision (ICD-10).

